# Dear Readers, Authors, Reviewers and Editorial Board Members of *Advances in Cognitive Psychology*

**DOI:** 10.5709/acp-0165-7

**Published:** 2015-03-31

## Abstract

In this letter from the editors, we wanted to inform you that our journal has been selected for coverage in Thomson Reuter’s products and services. Beginning with volume 8, issue 1, 2012, Advances in Cognitive Psychology (ACP) will be indexed and abstracted in:

•Social Sciences Citation Index

•Journal Citation Reports/Social Sciences Edition

•Current Contents/Social and Behavioral Sciences

This is of course very good news, and implies that we soon will receive an Impact Factor. As our journal is both an Open Access journal and charges up to now no publishing fees this may imply that the number of submissions increases.

We especially wanted to thank all the people who helped us in making this possible. First, and most important of all, we want to thank our good friend and colleague, Prof. dr. hab. Piotr Jakowksi. Piotr was the great inspirer of our journal. Many years ago, he came up with the idea of a pure electronic version of a new open access journal in the field of cognitive psychology, and after gathering people willing to be part of the editorial board, ACP was launched and the first volume got out in 2005. Piotr led the journal until his very unfortunate death in January 2011. Shortly thereafter, the decision was made by the editorial board members that the journal should get a chance to persist and it was decided that we (Rob and Ulrich) would try to do this by serving as the co-editor and the editor in chief, respectively. This, however, would not have been possible without support from the University of Finance and Management, in particular Krzysztof Godlewski, the financial director of the university, and also the help of Marcin Staniewski. Krzysztof continued to believe that it might be possible to successfully expand the impact of the journal, and due to this support we were finally able to get access to the ISI (Institute for Scientific Information) Master list of scientific journals. Obviously, this also would not have been possible without help from people from the IT department of Vizja that helped us with maintaining and renewing the website. Furthermore, several people were involved with improving the graphical design of the website, and also in developing flyers to promote ACP at national and international conferences.

A scientific journal cannot exist without an editorial manager. Several persons supported the journal in this role. We want to thank Anita Biauska, Magdalena Berkowska, and Jacek Grunt-Mejer for all their past work for ACP. During the last years, Jacek was of great help, especially when this involved solving more technical problems, and without him the transition to the new ACP website in 2014 would not have been possible. Other crucial technical support comes from Marlena Bartczak, who took care of the technical editorial work, and who is responsible for the nice layout of all that appeared in our journal. Due to maternity and parental leave her duties have temporarily been taken over by Dobrosia Jakowska, who was already involved in the implementation of hyperlinks. Thanks for all your work!

Special thanks go to all the members of our advisory and editorial board who continued to support us: Christian Buechel, Markus Kiefer, Isabelle Peretz, Ernst Poeppel, Bruno Repp, Ingrid Scharlau, Vincent Walsh, Elger Abrahamse, Gisa Aschersleben, Arndt Broeder, Claus-Christian Carbon, Simone Dalla Bella, Magosia Fajkowska, Anna Grabowska, Peter Keller, Iring Koch, Grzegorz Króliczak, Micha Kuniecki, Markus Lappe, Bernd Leplow, Severine Samson, Elisabet Service, Michael Spivey, Kate Stevens, Rolf Verleger, Martin Voracek, and Dirk Vorberg. As we indicated in our previous letters, we are also grateful for all the efforts that the reviewers put in evaluating submissions to our journal (see the lists on our website), and especially to the authors that submitted their papers to our journal.Thanks a lot. Without all your work this would not have been possible.

We decided that our letter concerning the year 2014 would come out in the first issue of 2015. In the last year, there were no major changes concerning our editorial board, but Iza Szumska was appointed as a new web-editor in December 2014. She replaces Jacek Grunt-Mejer.

Out of all of ACP’s submissions in the year 2014, one was withdrawn at a later stage, 8% were rejected without external review, 45% were rejected after external review, 12% were accepted, and the remaining manuscripts are currently being revised or are under review.

We would like to draw your attention to the Neuronus conference (http://neuronusforum.pl/). This conference will be held in Kraków from the 17th to the 19th of April 2015, and with this conference we have a special bound, which already resulted in a special issue devoted to Neuronus conferences in 2013. For the coming year, there is again the plan to organize a new special issue that may come out in 2016. More information will be available on the Neuronus conference in Kraków.

In the last year, there were no major changes in the journal’s budget. We are very grateful to the University of Finance and Management in Warsaw that continued to financially support ACP in the last year. Nevertheless, there is a possibility that we will have to ask authors for (voluntary) contributions to cover ACP’s publication costs, which include maintenance of the website, costs of cross-referencing, and costs for proof-editing and language corrections.

In conclusion of this letter, we kindly wanted to remind you that we continue to welcome your submissions to our journal. Also, should you have any questions or comments regarding this letter, or ACP, please feel always free to contact us.


Sincerely yours,

Rob van der Lubbe

Ulrich Ansorge


